# Plasma biomarkers for prognosis of cognitive decline in patients with mild cognitive impairment

**DOI:** 10.1093/braincomms/fcac155

**Published:** 2022-06-14

**Authors:** Pia Kivisäkk, Colin Magdamo, Bianca A Trombetta, Ayush Noori, Yi kai E Kuo, Lori B Chibnik, Becky C Carlyle, Alberto Serrano-Pozo, Clemens R Scherzer, Bradley T Hyman, Sudeshna Das, Steven E Arnold

**Affiliations:** Alzheimer’s Clinical & Translational Research Unit and Massachusetts Alzheimer’s Disease Research Center, Department of Neurology, Harvard Medical School, Massachusetts General Hospital, Charlestown, MA 02129, USA; Alzheimer’s Clinical & Translational Research Unit and Massachusetts Alzheimer’s Disease Research Center, Department of Neurology, Harvard Medical School, Massachusetts General Hospital, Charlestown, MA 02129, USA; Alzheimer’s Clinical & Translational Research Unit and Massachusetts Alzheimer’s Disease Research Center, Department of Neurology, Harvard Medical School, Massachusetts General Hospital, Charlestown, MA 02129, USA; Alzheimer’s Clinical & Translational Research Unit and Massachusetts Alzheimer’s Disease Research Center, Department of Neurology, Harvard Medical School, Massachusetts General Hospital, Charlestown, MA 02129, USA; Alzheimer’s Clinical & Translational Research Unit and Massachusetts Alzheimer’s Disease Research Center, Department of Neurology, Harvard Medical School, Massachusetts General Hospital, Charlestown, MA 02129, USA; Alzheimer’s Clinical & Translational Research Unit and Massachusetts Alzheimer’s Disease Research Center, Department of Neurology, Harvard Medical School, Massachusetts General Hospital, Charlestown, MA 02129, USA; Alzheimer’s Clinical & Translational Research Unit and Massachusetts Alzheimer’s Disease Research Center, Department of Neurology, Harvard Medical School, Massachusetts General Hospital, Charlestown, MA 02129, USA; Alzheimer’s Clinical & Translational Research Unit and Massachusetts Alzheimer’s Disease Research Center, Department of Neurology, Harvard Medical School, Massachusetts General Hospital, Charlestown, MA 02129, USA; Center for Advanced Parkinson Research and Precision Neurology Program, Harvard Medical School, Brigham and Women’s Hospital, Boston, MA 02115, USA; Alzheimer’s Clinical & Translational Research Unit and Massachusetts Alzheimer’s Disease Research Center, Department of Neurology, Harvard Medical School, Massachusetts General Hospital, Charlestown, MA 02129, USA; Alzheimer’s Clinical & Translational Research Unit and Massachusetts Alzheimer’s Disease Research Center, Department of Neurology, Harvard Medical School, Massachusetts General Hospital, Charlestown, MA 02129, USA; Alzheimer’s Clinical & Translational Research Unit and Massachusetts Alzheimer’s Disease Research Center, Department of Neurology, Harvard Medical School, Massachusetts General Hospital, Charlestown, MA 02129, USA

**Keywords:** prognostic biomarkers, mild cognitive impairment, Alzheimer's disease, plasma biomarkers

## Abstract

Plasma-based biomarkers present a promising approach in the research and clinical practice of Alzheimer's disease as they are inexpensive, accessible and minimally invasive. In particular, prognostic biomarkers of cognitive decline may aid in planning and management of clinical care. Although recent studies have demonstrated the prognostic utility of plasma biomarkers of Alzheimer pathology or neurodegeneration, such as pTau-181 and NF-L, whether other plasma biomarkers can further improve prediction of cognitive decline is undetermined. We conducted an observational cohort study to determine the prognostic utility of plasma biomarkers in predicting progression to dementia for individuals presenting with mild cognitive impairment due to probable Alzheimer's disease. We used the Olink™ Proximity Extension Assay technology to measure the level of 460 circulating proteins in banked plasma samples of all participants. We used a discovery data set comprised 60 individuals with mild cognitive impairment (30 progressors and 30 stable) and a validation data set consisting of 21 stable and 21 progressors. We developed a machine learning model to distinguish progressors from stable and used 44 proteins with significantly different plasma levels in progressors versus stable along with age, sex, education and baseline cognition as candidate features. A model with age, education, APOE genotype, baseline cognition, plasma pTau-181 and 12 plasma Olink protein biomarker levels was able to distinguish progressors from stable with 86.7% accuracy (mean area under the curve = 0.88). In the validation data set, the model accuracy was 78.6%. The Olink proteins selected by the model included those associated with vascular injury and neuroinflammation (e.g. IL-8, IL-17A, TIMP-4, MMP7). In addition, to compare these prognostic biomarkers to those that are altered in Alzheimer's disease or other types of dementia relative to controls, we analyzed samples from 20 individuals with Alzheimer, 30 with non-Alzheimer dementias and 34 with normal cognition. The proteins NF-L and PTP-1B were significantly higher in both Alzheimer and non-Alzheimer dementias compared with cognitively normal individuals. Interestingly, the prognostic markers of decline at the mild cognitive impairment stage did not overlap with those that differed between dementia and control cases. In summary, our findings suggest that plasma biomarkers of inflammation and vascular injury are associated with cognitive decline. Developing a plasma biomarker profile could aid in prognostic deliberations and identify individuals at higher risk of dementia in clinical practice.

## Teaser

Kivisäkk *et al*. report that in participants presenting with mild cognitive impairment, a plasma biomarker model was able to distinguish progressors with 86.7% accuracy. Study suggests that developing a plasma biomarker profile could aid in predicting disease progression and identify individuals at higher risk of dementia in clinical practice.

## Graphical Abstract

**Graphical abstract:**
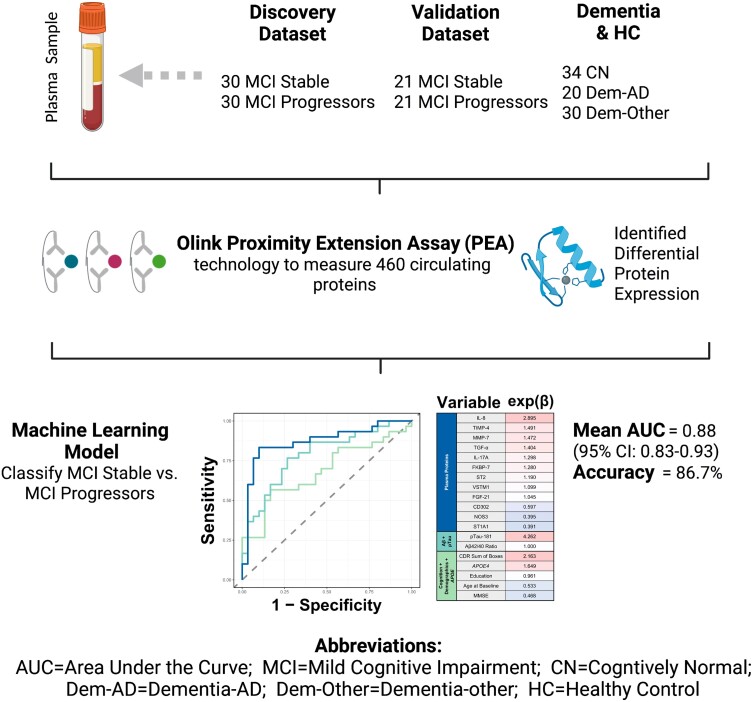

## Introduction

Mild cognitive impairment (MCI) is a transitional stage between the common cognitive decline of normal aging and the more serious decline into dementia. Individuals with MCI have evidence of cognitive impairment, but their independence in functional abilities is mostly preserved.^1^ MCI is a useful clinical diagnostic construct with prognostic implications because the likelihood of progression to dementia (i.e. a cognitive impairment that affects daily independent functioning) among people with MCI is greater than among cognitively normal individuals.^2^ However, because MCI is a heterogeneous condition, it is difficult to predict with accuracy if and when an individual with MCI will progress to dementia. Models for prediction of clinical MCI progression often include complex multiple domain neuropsychological testing, CSF biomarker analysis and/or neuroimaging.^3^ There is an urgent need for low cost, easily accessible and non-invasive alternatives such as blood-based biomarkers. Blood-based biomarkers of classic Alzheimer's disease pathology—β-amyloid (Aβ) and phospho-tau (pTau)—and neurodegeneration have improved in the last few years, with promise for establishing an ‘ATN’ diagnosis of Alzheimer's disease^4^ without need for cerebrospinal fluid testing or positron emission tomography (PET) neuroimaging. With regard to neurodegeneration, plasma neurofilament light polypeptide (NF-L, gene name *NEFL*) has been extensively studied and is associated with risk of developing Alzheimer's disease and non-Alzheimer's disease dementia,^5^ with Alzheimer's disease diagnosis, as well as positive Alzheimer's disease imaging biomarkers.^6^ However, the utility of blood-based ATN biomarkers in predicting MCI progression remains indefinite. Recent studies indicate that plasma or CSF levels of NF-L and various tau epitopes are elevated in individuals with MCI who progress to dementia,^7–10^ but this has not been consistently observed in other studies.^6,11,12^ Cullen *et al*. successfully used a combination of plasma levels of Aβ42/40, pTau-181 and NF-L along with age, sex and education for an individualized prognosis of MCI patients^10^ and achieved an AUC of 0.88 in their study.

While great progress has been recently made in plasma biomarkers of Aβ and pTau, it is also clear that the pathophysiology of Alzheimer's disease is not limited to the Aβ cascade and tauopathy and that processes such as inflammation, vascular injury, oxidative injury and disruption of metabolic pathways may also contribute to the progression of the disease but are not reflected by changes in the classic Alzheimer's disease biomarkers or the ATN classification scheme. It is therefore likely that combinations of blood-based protein biomarkers reflecting different pathways will increase the sensitivity and specificity of a single biomarker test to predict MCI-to-dementia conversion,^13^ and efforts have been made to establish multivariate biomarker panels that can predict clinical progression of MCI. Some of these studies have reported high accuracy in predicting MCI conversion to dementia,^14–16^ but these biomarkers were not combined with the ATN biomarkers. In addition, other studies using the same panels failed to reproduce such high accuracy in the context of classifying MCI and/or dementia,^17–20^ and it is not well-established which biomarkers are most informative.

Here, we used a novel, highly sensitive and specific multiplex immunoassay^21^ to measure plasma levels of 460 protein biomarkers selected to reflect a range of pathophysiological processes implicated in Alzheimer's disease with a focus on inflammation, metabolism, vascular injury and neurodegeneration, in two data sets of 60 (discovery) and 42 (validation) individuals to determine the prognostic utility of plasma biomarkers. We hypothesized that a combination of plasma biomarkers, as well as *APOE* genotype, plasma Aβ42/40 and pTau-181 would predict MCI progression to Alzheimer's disease dementia within 5 years with improved accuracy compared with demographic and clinical variables alone. The secondary objective of the study was to compare these prognostic biomarkers to those that are altered in Alzheimer's disease or other types of dementia relative to controls.

## Materials and methods

### Study participants

Plasma samples were obtained from participants in the Massachusetts Alzheimer's Disease Research Center (MADRC) longitudinal cohort of cognitive aging between 2008 and 2015. The MADRC is one of the National Institute on Aging (NIA)-funded Alzheimer's Disease Research Centers. Annual assessments include biofluid collection, a general and neurological examination, a semi-structured interview with the participant and/or informant to record cognitive symptoms and score the Clinical Dementia Rating scale (CDR^®^ Dementia Staging Instrument), and a comprehensive battery of neuropsychological tests.^22^ Plasma samples drawn at visit from the participants were banked by the Harvard Biomarkers Study.^23^ The study was approved by the Mass General Brigham Institutional Review Board (2006P002104), and all participants provided written informed consent. Cognitive status was determined at each approximately annual visit by a consensus team after a detailed examination and review of all available information according to 2011 National Institute on Aging-Alzheimer's Association (NIA-AA) diagnostic criteria for mild cognitive impairment (MCI)^1^ and for Alzheimer's disease.^24^ Diagnosis of frontotemporal dementia, Lewy body disease, or progressive supranuclear palsy were also similarly made according to defined standards.^25–27^ CSF and imaging biomarkers were available only for a subset of the participants.

Specifically, samples from several groups were selected (sample sizes were guided by previous studies):

Discovery samples: The discovery samples were drawn at baseline from 60 participants with a clinical diagnosis of MCI due to probable Alzheimer's disease, global CDR score of 0.5 and at least five annual follow-up visits. The participants were classified into two groups based on their global CDR trajectory over a 5-year follow-up period: ‘MCI-progressors’, if global CDR score increased from 0.5 to 1 (i.e. participants who converted from MCI to dementia in ≤5 years) and ‘MCI-stable’, if no change in global CDR score (see Table 1A).
Validation samples: The validation samples were drawn from 42 additional MCI participants with baseline clinical diagnosis of MCI due to probable Alzheimer's disease, baseline global CDR score of 0.5 and at least five annual follow-up visits with unchanged CDR scores (MCI-stable) or declined in ≤5 years (MCI-progressors). Records were thoroughly reviewed to exclude participants with ambiguous clinical presentation at first and/or follow-up visits (see Table 1B).
Dementia and heathy control (HC) samples: To compare prognostic biomarkers with diagnostic biomarkers, we selected three other groups: (i) Dementia-Alzheimer's disease (Dem-Alzheimer's disease): 20 participants with a global CDR score ≥1 and a clinical aetiologic diagnosis of dementia due to probable Alzheimer's disease
(ii) Dementia-other (Dem-Other): 30 participants with a global CDR score ≥1 and a clinical aetiologic diagnosis of non-Alzheimer's disease dementia such as frontotemporal dementia, Lewy body disease, or progressive supranuclear palsy
and (iii) Cognitively normal (CN): 34 age- and sex-matched healthy participants with normal neuropsychological testing scores and no subjective cognitive symptoms over 5 + years (see Table 1C).

### Plasma biomarker measurements

Samples were collected in K2EDTA tubes, centrifuged at 2000 g for 5 min, frozen in low retention polypropylene cryovials within 4 h of collection and stored at −80°C until use. Samples were processed and frozen within 4 h from blood draw and stored at −80°C until use. Plasma samples were then sent to Olink Analysis Services (Olink Proteomics, Watertown, MA) and plasma levels of 460 protein biomarkers were measured by Proximity Extension Assay (PEA) technology^21^ using five panels (Immuno-Oncology, Neuro-Exploratory, Cardiovascular III, Inflammation and Cardiometabolic). This is a novel technology that combines antibody-epitope recognition and binding with quantitative polymerase chain reaction (qPCR). Briefly, circulating plasma proteins are specifically bound by DNA-tagged antibodies upon epitope recognition. Pairs of complementary hybridized DNA tags are then amplified via qPCR. Data were pre-processed using the Olink NPX Manager software and presented as Normalized Protein Expression (NPX) values. Samples from the discovery and non-MCI groups were evenly distributed across six plates as part of a larger sample cohort, whereas all the validation samples were run on one plate. Three quality control (QC) samples were included on all plates to evaluate intra- and inter- plate variability. In total, 60 proteins did not pass QC thresholds, either because more than five samples were below the lower limit of detection (LLOD, *n* = 57) or because they had an inter-plate coefficient of variation (CV) > 50% (*n* = 3) and were removed from further analyses. Some proteins were represented on multiple panels, allowing further inter-assay consistency checks, and these were all excellent (correlation ≥ 0.8). If a protein was represented on multiple panels, we included only one randomly chosen instance. In total, 362 proteins were entered into statistical analyses. Technical validation of CX3CL1, IL-8, and CSF-1 plasma levels were performed using multiplexed U-plex immunoassays from Meso Scale Diagnostics (Rockville, MD) and acquired on a MESO QuickPlex SQ129 reader (Meso Scale Diagnostics, MSD) following manufacturer's instructions. Plasma levels of Aβ_40_ and Aβ_42_ were measured using Euroimmun Beta-Amyloid (1-40) and (1-42) ELISA assays (Lübeck, Germany) performed on a semiautomated TECAN Freedom EVO liquid handler (Männedorf, Switzerland) following manufacturer's instructions and the Aβ42/40 ratio calculated. pTau-181 plasma levels were measured using the Quanterix Simoa pTau-181 V2 Advantage Kit on a fully automated Quanterix HD-X analyzer (Billerica, MA).

### Statistical analysis

All statistical analyses were conducted using R version 4.0.2. Continuous variables were centred and scaled for all analyses. Missing data for plasma biomarkers was imputed as the mean of available data points. Differential expression analysis of MCI-progressor versus MCI-stable, Dem-Alzheimer's disease versus CN, and Dem-Other versus CN was conducted using the *limma* package in R,^28^ which uses empirical Bayes to borrow information across all proteins and increase power, with Olink's protein NPX values as the outcome, and diagnostic group, age and sex as predictors. Multiple comparisons were adjusted using the Benjamini-Hochberg method and a threshold of FDR adjusted *P*-value (or *q* value) of 0.05 was used. Correlation between MSD and Olink PEA assays were computed using the R *cor* function. All plots were generated using the R *ggplot2* package.

We used the least absolute shrinkage and selection operator method (LASSO),^29^ a supervised machine learning model, to classify MCI participants into MCI-progressors versus MCI-stable (classes specified by the CDR trajectory as described previously). We developed three models: Model 1 was trained with baseline cognitive scores—Mini-Mental State Examination (MMSE),^30^ Functional Assessment Questionnaire (FAQ),^31^ and CDR Sum of Boxes (CDR-SOB)—age, sex, education and *APOE* genotype. *APOE* genotype was coded as 1 for ε4 carriers (ε4/ε4, ε3/ε4 and ε2/ε4) and 0 for ε4 non-carriers. Model 2 also included plasma Aβ42/40 and pTau-181 levels. Model 3 was trained with all the variables of Model 2 and the nominally significant plasma proteins resulting from the differential expression analyses. Model training and 5-fold cross-validation were performed on the discovery samples using the *glmnet* package in R and the value of the LASSO regularization parameter lambda was selected by maximizing the mean receiver operating characteristic (ROC) area under the curve (AUC) across the five folds. The optimal lambda was used to enforce a constraint on the sum of the magnitudes of the estimated coefficients. This constraint was used to prevent overfitting and perform variable selection. Results from the LASSO models are presented as the mean and 95% confidence interval (CI) of the cross-validated AUC. The specificity, sensitivity and other measures at the selected lambda value are reported. The performance of the trained model (Model 3) was then evaluated on the validation samples.

### Data availability

All protein concentration (NPX) data from the Olink^™^ panels are available in Supplementary Table 1.

## Results

### Characteristics of study participants

**Figure 1:**
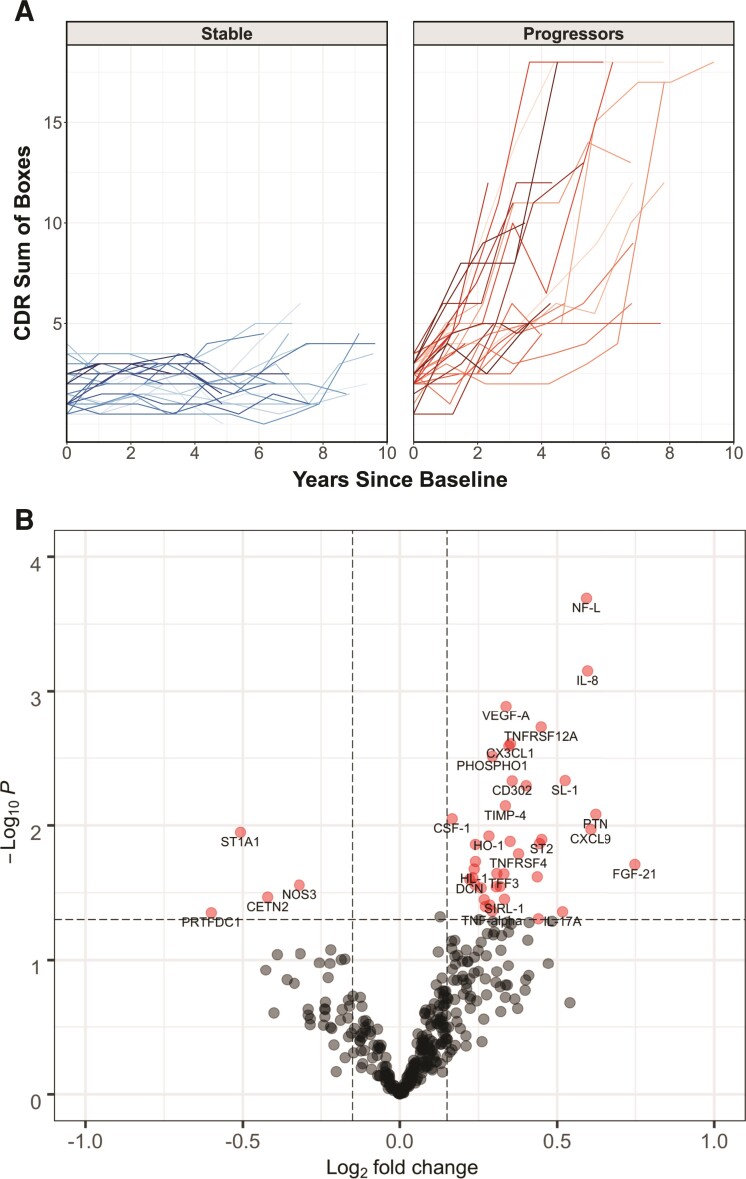
**MCI-progressors versus stable.** (**A**) Trajectory of CDR Sum of Boxes scores over longitudinal visits in MCI-stable and progressors. (**B**) Differential expression of proteins in MCI-progressors versus stable. Proteins with a *P* < 0.05 and fold-change >10% are displayed (total 44 proteins)

In the discovery data set, the MCI-progressors and MCI-stable participants were 75.9 ± 9.1 and 78.1 ± 6.7 years old (mean ± SD), respectively, and 50.0% of participants in each group were female (Table 1A). The MCI-progressors had an average bassline MMSE of 26.2 ± 4.3 and 56.7% had a college education or more, while MCI-stable had an average baseline MMSE of 28.3 ± 1.4 and 66.7% had a college education or more. The baseline CDR-SOB, baseline MMSE and baseline ptau-181 were significantly different (*P* < 0.05) between the MCI-stable and the MCI-progressors. The global CDR was 0.5 at baseline for both groups. Fig. 1A illustrates the CDR-SOB trajectory for the 30 MCI-progressors and 30 MCI-stable participants from the discovery data set, with an increase in the global CDR score from 0.5 to 1 between years 2 and 5 in the MCI-progressors and the stability of the global CDR score in the MCI-stable group (as defined by our study design).

Table 1B and C display the characteristics of the validation and the dementia and HC data sets, respectively. In the validation data set, like the discovery data set, the baseline CDR-SOB, baseline MMSE, and baseline ptau-181 were significantly different (*P* < 0.05) between the MCI-stable and the MCI-progressors. In addition, the number of participants with a college education or more was significantly higher in the MCI-stable group in this data set. In the dementia and HC data set, the proportion of *APOE* ε4 carriers (*APOE* genotype was missing for oneparticipant) and CDR Sum of Boxes was significantly higher (*P* < 0.05) in the Dem-Alzheimer's disease group compared with those with normal cognition. For the Dem-Other group, the baseline age and CDR Sum of Boxes was significantly different (*P* < 0.05) compared with those with normal cognition.

### Differentially expressed plasma biomarkers between MCI-progressors and MCI-stable

Plasma biomarkers were analyzed using Olink PEA in the discovery samples drawn at baseline. This methodology based on DNA-tagged antibodies, and quantitative PCR amplification of pairs of hybridized DNA tags enables the simultaneous measurement of a customized panel of hundreds of plasma proteins with high sensitivity and specificity.^21^ The levels of the 362 plasma biomarkers passing our stringent QC criteria (i.e. Olink NPX values) for all samples analyzed with the diagnostic group, age and sex are available in Supplementary Table 1.

In an exploratory analysis to identify putative biomarkers associated with clinical progression from MCI to Alzheimer's disease dementia, we determined differentially expressed proteins between the two MCI groups. A total of 44 proteins were significantly altered in MCI-progressors versus MCI-stable (*P* < 0.05), controlling for age, sex and baseline CDR-SOB (Fig. 1B, Supplementary Table 2). None of these were significant after multiple comparison corrections. These 44 differentially expressed proteins were all increased in MCI-progressors versus MCI-stable, except for PRTFDC1, ST1A1, CETN2 and NOS3, which were decreased. The proteins were primarily representative of inflammatory/chemotaxis (CCL23, CSF-1, CX3CL1, CXCL9, IL-8, LTBR, MCP-1, ST2, TNFRSF12A), extracellular matrix (MMP-3, PTN and TIMP-4), neurodegeneration (NF-L) and vascular processes (PGF, MB and VEGFA). The metabolic biomarker PHOSPHO1—an enzyme that dephosphorylates phospholipids—was also significantly increased in MCI-progressors versus MCI-stable participants. Regarding the magnitude of the differences, five proteins had an effect size of >50%: NF-L (50.8% increase in MCI-progressors versus MCI-stable), IL-8 (53.0% increase), CXCL9 (52.3% increase), PTN (54.0% increase) and FGF-21 (68.0% increase). When performing the same analysis in the validation samples, six of the original 44 proteins were elevated in MCI-progressors versus MCI-stable (unadjusted *P* < 0.05): MCP-1, PTN, CSF-1, TGF-alpha, FGF-21 and CX3CL1.

**Figure 2:**
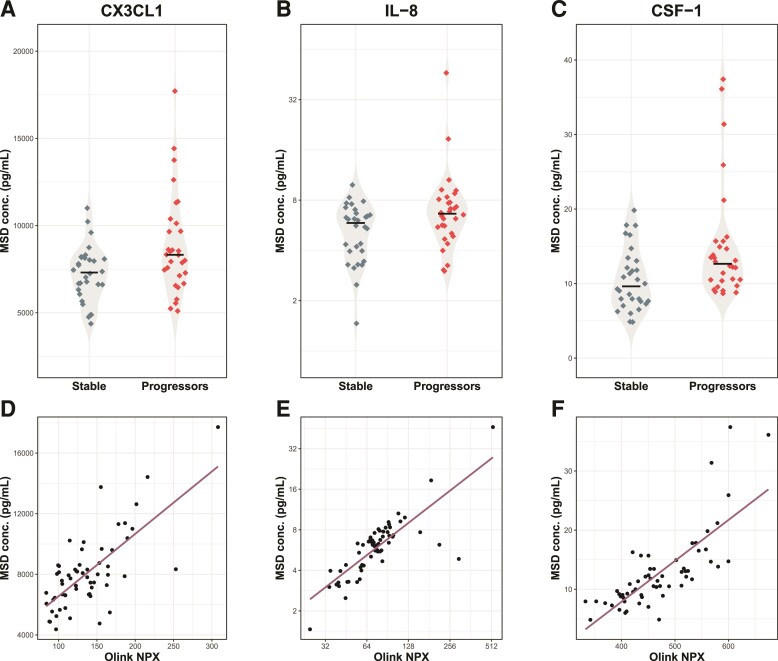
**Technical validation of Olink PEA results.** (**A**–**C**) Comparison of protein concentrations between MCI-progressors and stable groups using immunoassays from MSD in additional vials of the same samples used for Olink PEA. Differences between the MCI-stable and MCI-progressor groups for (**A**) CX3CL1 (*t* = 2.76, *P* = 0.008), (**B**) IL-8 (*t* = 2.77, *P* = 0.008) and (**C**) CSF-1 (*t* = 2.77, *P* = 0.008) were all significant. (**D–F**) Pearson correlation between Olink PEA and MSD assays (*r*: CX3CL1 = 0.5 IL-8 = 0.7 and CSF-1 = 0.6). Note that the concentration of IL-8 is in logarithmic scale

Next, to validate the results of the Olink PEA against other commonly used platforms, we performed a technical validation for the differentially expressed proteins CX3CL1, IL-8 and CSF-1 using electrochemiluminescence immunoassays (MSD) with additional vials of the same samples used for the Olink PEA assays. Differences between the MCI-stable and MCI-progressor groups remained statistically significant for all three proteins (*P* < 0.05 for all comparisons Fig. 2A–C). Biomarker levels measured with MSD and PEA assays were also significantly correlated, with *R*^2^ values between 0.5 and 0.7 (Fig. 2D–F). In addition, we observed excellent correlation between plasma levels of NF-L measured by the Olink PEA technology and Quanterix Simoa (*R*^2^ = 0.91, *P* < 0.05 Supplementary Fig. 1).

### A machine learning model including plasma protein biomarkers classifies MCI-progressors versus MCI-stable with high accuracy

**Figure 3:**
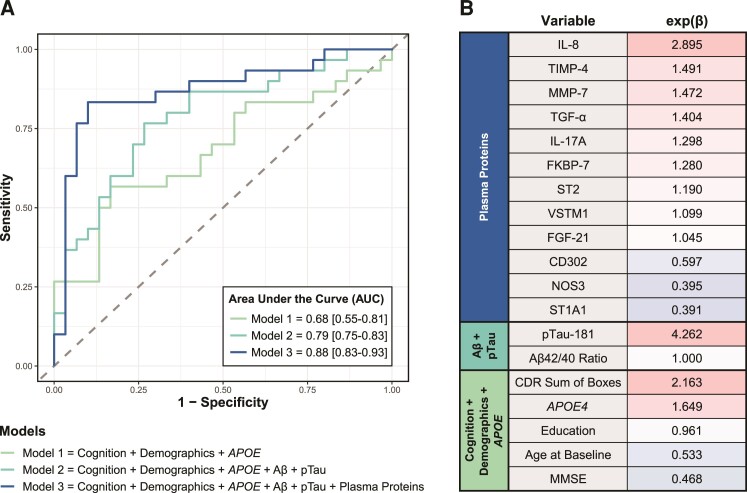
**Machine learning model to discriminate MCI-progressors from stable.** (**A**) ROC curves for (i) *Model 1* with candidate variables: baseline cognitive measures, age, sex, education and *APOE* genotype (AUC = 0.68, 95% CI: 0.55-0.81) (ii) *Model 2* with candidate variables: baseline cognitive measures, age, sex, education, *APOE* genotype, plasma Aβ42/40 ratio and plasma pTau-181 (AUC = 0.79, 95% CI: 0.75-0.83) and (iii) *Model 3* with candidate variables: baseline cognitive measures, age, sex, education, *APOE* genotype, plasma Aβ42/40 ratio, plasma pTau-181, and Olink plasma proteins (AUC = 0.88, 95% CI: 0.83-0.93). (**B**) Heatmap of the standardized beta coefficients [exp(β)] of the variables selected by LASSO regularization in Model 3. A total of 12 plasma proteins, plasma pTau-181, CDR Sum of Boxes, *APOE* genotype, education, age at baseline and MMSE at baseline, were selected in Model 3. All continuous variables were centred and scaled for these analyses

We next investigated whether a machine learning model could discriminate MCI-progressors from MCI-stable individuals using the Olink biomarkers associated with decline in the exploratory analysis. We reasoned that our large multidimensional data with demographic, cognitive and *APOE* genotype data, as well as plasma protein levels provided an opportunity to develop a machine learning algorithm to classify MCI individuals into MCI-stable versus MCI-progressors and, thus, predict progression from MCI to dementia. To explore the relative contribution of the Olink biomarkers, we trained three regularized multivariate logistic regression models with increasing number of inputs and compared them to each other. The first model (Model 1) had the following candidate variables: baseline cognitive measures (MMSE, FAQ and CDR-SOB), demographic variables (age, sex and education) and *APOE* genotype. This model classified participants into the two groups with 70% accuracy (AUC = 0.68, 95% CI: 0.55–0.81; Specificity = 80% and Sensitivity = 57% at the threshold of maximum accuracy; Fig. 3A). The second model (Model 2) added plasma measures of Aβ42/40 and pTau-181 to the candidate variables of Model 1 and further improved the performance (mean AUC across five cross-validation folds = 0.79, 95% CI: 0.75–0.83 specificity = 73% and sensitivity = 76% at the threshold of maximum accuracy; Fig. 3A). The third model (Model 3) that added the scaled and centred levels of 44 proteins as candidate variables to those of Model 2 substantially improved model performance [mean AUC = 0.88 95% CI: 0.83–0.93; specificity = 90% and sensitivity = 83.3% at the threshold of maximum accuracy (86.7%)

Fig. 3A and was chosen as the final model. Model 3 selected 12 of the 44 differentially expressed proteins in addition to age at baseline, education, baseline CDR-SOB, baseline MMSE, *APOE* genotype and pTau-181 as contributors to this classification task (ranked in Fig. 3B). The strongest predictor was plasma pTau-181 levels (odds of progression increases 4.26-fold with 1σ (1.5 pg/ml) change in pTau-181 levels), which is concordant with other studies showing similar results.^9,10^ Of note, plasma Aβ42/40 and NF-L were not selected by the model as predictors.

To test the performance of our final machine learning algorithm (Model 3), we applied it to a validation data set with samples from 21 MCI-progressors and 21 MCI-stable participants selected from the MADRC longitudinal cohort (Table 1B). The final regularized logistic regression Model 3, which selected baseline age, education, *APOE* genotype, baseline cognitive scores (MMSE, and CDR-SOB), and the plasma levels of pTau-181, as well as 12 other proteins as predictors (see Fig. 3B), was able to classify MCI-progressors versus MCI-stable participants with an overall accuracy of 78.6% (AUC = 0.83; sensitivity = 0.81 and specificity = 0.76 at the threshold of maximum accuracy). The AUC was lower than that of the discovery data set, suggesting modest generalizability beyond the training data set.

### Biomarkers of MCI progression to Alzheimer's disease dementia are distinct from biomarkers of Alzheimer's disease dementia

Next, we asked whether these prognostic biomarkers of progression from MCI to dementia overlap with diagnostic biomarkers of Alzheimer's disease and other types of dementia. To this end, we analyzed the 362 Olink PEA protein biomarkers that passed QC criteria in plasma samples from dementia and healthy control groups: Alzheimer's disease dementia (Dem-Alzheimer's disease, *n* = 20), dementia of non-Alzheimer's disease aetiology (Dem-Other, *n* = 30), and CN participants (CN, *n* = 34) (see Methods section and Table 1).

**Table 1:** Study participant summary statistics

Characteristics	MCI-stable		MCI-progressors
Discovery data set			
Number of participants (*N*)	30		30
Age [Mean (SD)]	75.9 (9.1)		78.1 (6.7)
Female [*N* (%)]	15 (50)		15 (50)
Follow-up time [median (min, max)]	6.6 (1.7, 10.5)		4.3 (1.1, 9.4)
College educated [*N* (%)]	20 (66.7)		17 (56.7)
APOE ε4 carriers [*N* (%)]	11 (36.7)		16 (53.3)
CDR sum of boxes [Mean (SD)]	1.8 (1)		2.4 (0.8)*
MMSE [Mean (SD)]	28.3 (1.4)		26.2 (4.3)*
Aβ42/40 [Mean (SD)]	0.1 (0)		0.1 (0)
pTau-181 [Mean (SD)]	2.1 (1)		3.6 (1.6)*
*N*-FL [Mean (SD)]	5 (0.6)		5.3 (0.7)
Validation data set			
Number of participants [*N*]	21		21
Age [Mean (SD)]	75.5 (7.7)		78.3 (8.2)
Female [*N* (%)]	8 (38.1)		13 (61.9)
Follow-up time [Median (Min, Max)]	9.0 (2.1, 11.6)		3.7 (5.3, 12.7)
College educated [*N* (%)]	17 (81.0)		15 (71.4)*
APOE ε4 carriers [*N* (%)]	4 (19.0)		12 (57.1)
CDR sum of boxes [Mean (SD)]	1.5 (0.9)		2.1 (1.1)*
MMSE [Mean (SD)]	28.5 (1.0)		27 (1.6)*
Aβ42/40 [Mean (SD)]	0.1 (0.0)		0.1 (0.1)
pTau-181 [Mean (SD)]	1.7 (0.9)		3.9 (1.9)*
*N*-FL [Mean (SD)]	4.8 (0.5)		5.1 (0.6)

Characteristics	CN	Dem-Alzheimer's disease	Dem-Other
Dementia and HC			
Number of participants [*N*]	34	20	30
Age [Mean (SD)]	76.2 (9.4)	78.3 (7.5)	69.9 (9.3)
Female [*N* (%)]	18 (52.9%)	10 (50.0%)	15 (50.0%)
Follow-up time [Median (Min, Max)]	8.7 (2.7, 11.5)	5.5 (2.1, 10.6)	3.9 (0.9, 9.6)
College educated [*N* (%)]	27 (79.4)	15 (75)	21 (70)
APOE ε4 Carriers [*N* (%)]	10 (29.4)	13 (65)*	6 (20)
CDR sum of boxes [Mean (SD)]	0 (0.1)	6.3 (2.6)^a^	3.7 (3.5)

Discovery data set consisted of 60 MCI (29 MCI-progressors, 31 MCI-stable). Validation data set consisted of 42 MCI (21 MCI-progressors and 21 MCI-stable), and dementia and healthy control (HC) data set included 34 cognitively normal (CN), 20 Alzheimer's disease Dementia (Dem-Alzheimer's disease), and 30 other dementia (Dem-Other) participants. Statistical comparisons were performed with Student’s *t*-test for continuous variables and χ^2^ for dichotomous variables.

* *P* < 0.05 for MCI-progressors versus MCI-stable groups.

**Figure 4:**
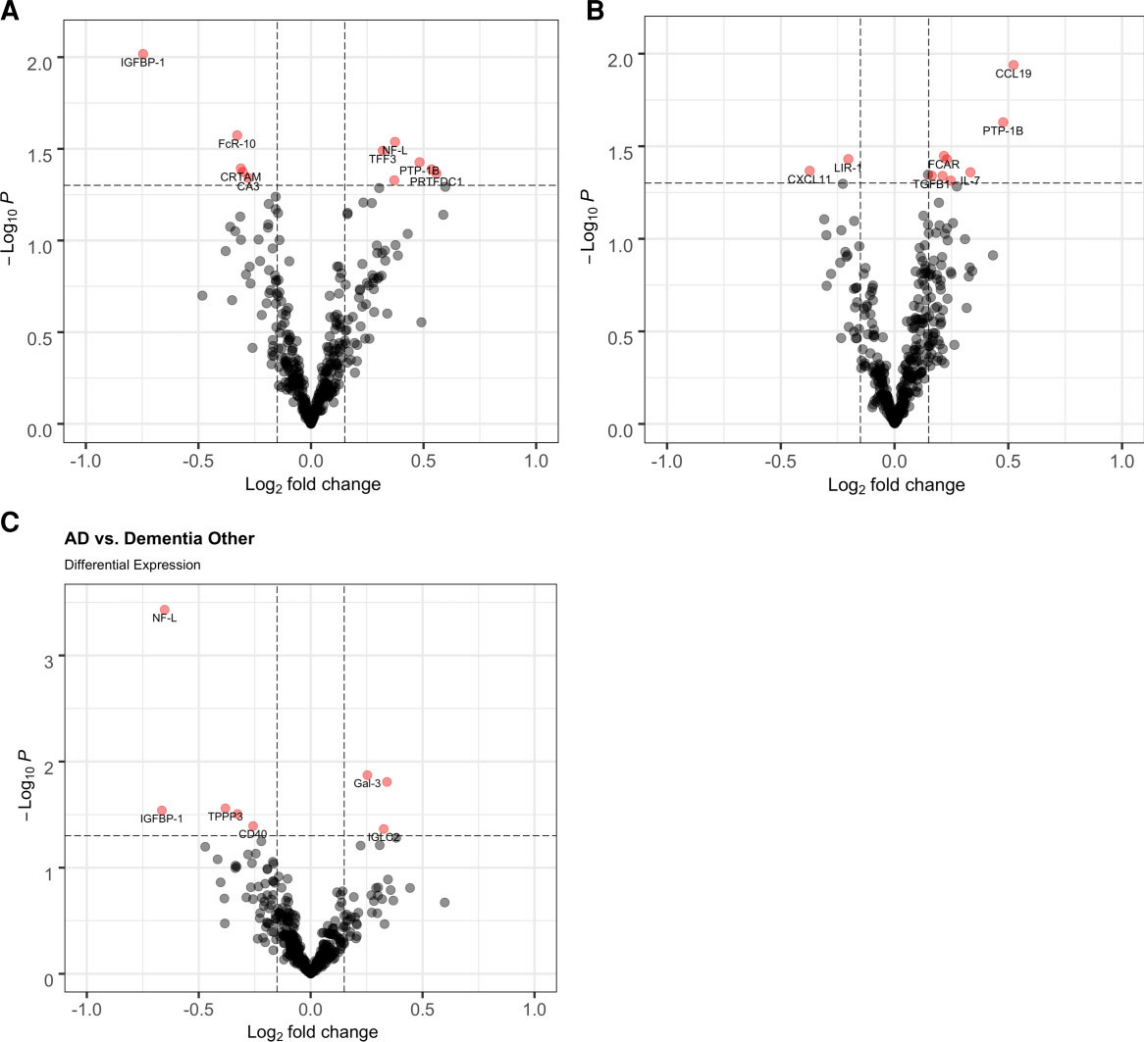
**Differential expression analysis of Alzheimer's disease and other dementia versus CN participants.** (**A**) Alzheimer's disease (AD) versus CN participants. (**B**) Dem-Other versus CN (C) AD vs Dem-Other participants. Proteins with a *P* < 0.05 and fold-change >10% are displayed. NF-L was significantly increased in Dem-Other versus CN (fold-change = 2.03, *P* = 4.43 × 10^−10^) but is not shown on the plot to keep the scales between the plots consistent.

Eleven proteins had altered (*n* = 6 increased and *n* = 5 decreased) plasma levels in Dem-Alzheimer's disease compared with CN (*P* < 0.05) (Fig. 4A, Supplementary Table 3A). Twelve proteins were altered (*n* = 10 increased and *n* = 2 decreased) in Dem-Other compared with CN (*P* < 0.05), of which NF-L was statistically significantly increased even after multiple comparisons (Fig. 4B, Supplementary Table 3B). There was little overlap between Dem-Alzheimer's disease and Dem-Other biomarkers; only NF-L and PTP-1B were common to both groups. Consistent with its known association with multiple neurodegenerative diseases,^6,8,32^ NF-L was highly increased in Dem-Other (fold-change = 2.03, *P* = 4.43 × 10^−10^) and moderately in Dem-Alzheimer's disease (fold-change = 1.29, *P* = 0.029) versus CN participants. PTP-1B, a regulator of insulin and leptin signalling that has been shown to be involved in multiple Alzheimer's disease processes,^33^ was moderately increased in both Dem-Alzheimer's disease (fold-change = 1.39, *P* = 0.037) and Dem-Other (fold-change = 1.39, *P* = 0.023) compared with CN individuals. Interestingly, only one of the proteins that was increased in MCI-progressors versus MCI-stable groups, NF-L, was also differentially expressed in Dem-Alzheimer's disease versus CN participants, indicating that prognostic biomarkers of progression from MCI to dementia due to probable Alzheimer's disease are not necessarily diagnostic biomarkers for dementia due to probable Alzheimer's disease. We also compared the Dem-Alzheimer's disease samples to the Dem-Other samples (Fig. 4C and Supplementary Table 3C). NF-L was significantly higher in the Dem-Other group compared to Dem-Alzheimer's disease (fold-change = 1.57, *P* = 0.0004). In addition, IGFBP-1, which was significantly lower in Dem-Alzheimer's disease compared with CN individuals, was also significantly lower in Dem-Alzheimer's disease compared with Dem-Other (fold-change = 0.63, *P* = 0.03), suggesting the change maybe unique to Alzheimer's disease pathology. Since the Dem-Alzheimer's disease group is significantly older than the Dem-Other group, the difference may also be age related.

## Discussion

Our large plasma biomarker panel revealed that biomarkers of inflammation/chemotaxis (CCL23, CX3CL1, CSF-1, CXCL9, IL-8 and TNFRSF12A), extracellular matrix remodelling (MMP-3 and TIMP-4), endothelial injury (NOS3 and VEGF-A), insulin-like growth factor signalling regulation (IGFBP2), and lipid metabolism (PHOSPHO1), in addition to neurodegeneration (NF-L), were associated with conversion of MCI to dementia within the probable Alzheimer's disease spectrum. Including a selection of these plasma proteins in our machine learning algorithm improved the prognostic accuracy not only of the simpler model including *APOE* genotype, cognitive measures, and demographic variables but also that of the model that additionally included plasma Aβ42/40 and pTau-181 measures.

There are several implications of these findings. First, they indicate that plasma biomarkers may be able to detect correlates of heterogeneity in the pathophysiological processes underlying cognitive impairment in the elderly. Moreover, these heterogeneous processes appear to be present only earlier in the disease process and not after a full dementia clinical phenotype is present. Akin to the interpretation that altered levels of plasma Aβ or pTau reflect their respective depositions in the brain, and that NF-L levels are a proxy for neuroaxonal degeneration, one possibility is that increased plasma level of the colony stimulating factor-1 (CSF-1) might reflect microglia activation, whereas the increased plasma levels of chemokines CCL23, CX3CL1 and CXCL9 might reflect the migration of these microglia towards amyloid plaques, neurofibrillary tangles and dying neurons. The fact that these biochemical alterations, known to occur in the Alzheimer's disease brain, are detectable in plasma could be explained by a disruption of the blood–brain barrier. Indeed, the endothelial nitric oxide synthase 3 (NOS3) was reduced by 27% while the vascular endothelial growth factor A (VEGF-A) was increased by 20% in MCI-progressors versus MCI-stable participants, suggesting that endothelial cell injury and angiogenesis are early phenomena.^34,35^ In addition, the higher levels of the metalloprotease stromelysin-1 (SL-1) and metalloprotease inhibitor 4 (TIMP-4) in MCI-progressors versus MCI-stable participants would have opposing effects on the integrity of collagen and other components of the extracellular matrix and capillary basement membranes, further reinforcing the notion that the blood–brain barrier might be disrupted and leaky early in the progression to dementia.^36^ Alternatively, the increased plasma levels of CSF-1, CCL23, CX3CL1 and CXCL9 we observed might represent a systemic immune dysregulation detectable in blood in parallel to immune processes within CNS. Peripheral immune dysregulation may be independent and only parenthetically related to Alzheimer's disease.^37^ Similarly, the alterations in endothelial and extracellular matrix plasma biomarkers might partly reflect peripheral changes, however these pathways have been highlighted by previous large-scale plasma/CSF biomarker and brain proteomic studies.^38,39^

Second, these results suggest that a multiplexed plasma biomarker panel like the one used here, with further validation, could provide useful prognostic information in a clinical setting by profiling various pathogenic cascades that might occur upstream, downstream, or in parallel to Aβ, pTau and neurodegeneration. Indeed, we show that applying a machine learning algorithm on such a multidimensional biomarker data set improves clinical prognosis, which remains an unmet need of patients at the MCI stage and their families. Of note, although other studies have identified markers of inflammation,^15,16,20^ there is little overlap with the proteins identified in our study. Similarly, the extracellular matrix proteins identified by our study are distinct from those reported by Yang *et al*.'s proteomics study.^14^ However, since the pathways identified in various studies are similar (even though the individual proteins are different), perhaps a large number of combined markers can be used to develop robust validated prognostic biomarkers.

Third, such panels could inform clinical trials,^40^ especially those targeting vascular or inflammatory risk factors^41^ and those testing multimodal lifestyle interventions.^42^ This approach may also enable the field to move closer to personalized medicine where it can be envisioned that MCI individuals will be enrolled in trials based on their biomarker profile of different pathophysiological processes involved in neurodegeneration. Further, the sample size required for a clinical trial can be reduced by enriching the trial with participants who are more likely to decline in the next few years. Indeed, using simulations Cullen *et al*. demonstrated that a combination of plasma Aβ42/40, pTau-217 and NF-L could potentially reduce the sample size of trials with cognition as primary endpoint by as much as 70%.^43^

Neuroimaging and CSF biomarkers have also been successfully used to predict disease progression.^44^ For example, baseline brain MRI measures, such as cortical thinning has been associated with a faster decline.^45^ Another study used a combination of neuropsychological tests, hippocampal volume and the CSF markers Aβ42, P-tau and T-tau to predict conversion from MCI to dementia (AUC = 0.96).^46^ Yet another applied a deep learning model to baseline neuroimaging biomarkers and longitudinal CSF and cognitive testing to predict conversion from MCI to probable Alzheimer's disease (AUC = 0.86).^47^ While neuroimaging and CSF prognostic biomarkers may achieve similar or better performance, blood-based biomarkers have the distinct advantage of low costs and higher accessibility.

Strengths of this study include the multiplexed quantification of 362 protein biomarkers to characterize an array of diverse functional alterations known to play a role in Alzheimer's disease pathophysiology; the careful selection of clinically characterized MCI, Dem-Alzheimer's disease, Dem-Other and CN samples from a longitudinal cohort study with long follow-up time the use of discovery and validation samples; and the application of machine learning models to find predictors of MCI progression to Alzheimer's disease dementia. Some limitations should also be acknowledged. The small sample size limits the generalizability of our results and warrants confirmation in larger, more diverse and independent external cohorts. Use of dementia-related medications was not considered in the analyses. Some of the participants may have been misclassified due to the lack of Alzheimer's disease CSF, PET or autopsy confirmation of Aβ and pTau pathologies. Finally, comorbid pathologies (i.e. vascular, Lewy body, TDP-43) may contribute to the progression from MCI to dementia but likely were not fully accounted for by the biomarkers studied.

In summary, our multiplexed plasma biomarker panel highlights inflammation/chemotaxis, extracellular matrix remodelling and vascular injury as early phenomena in Alzheimer's disease pathophysiology predicting the progression of MCI to dementia due to probable Alzheimer's disease. These plasma biomarker panels may provide useful prognostic information in a clinical setting and offer an opportunity for personalized medicine strategies beyond Aβ and tau-directed therapies.

## Supplementary Material

fcac155_Supplementary_Data

## Abbreviations

Aβ =: β-amyloid
ATN =: β-amyloid/tau/neuronal damage
AUC =: area under the curve
CDR =: Clinical Dementia Rating
CDR-SOB =: CDR Sum of Boxes
CI =: confidence interval
CN =: cognitively normal
CV =: coefficient of variation
Dem-Alzheimer's disease =: Dementia-Alzheimer's disease
Dem-Other =: Dementia-other
FAQ =: Functional Assessment Questionnaire
HC =: healthy control
LASSO =: least absolute shrinkage and selection operator method
LLOD =: lower limit of detection
MADRC =: Massachusetts Alzheimer's Disease Research Center
MCI =: mild cognitive impairment
MMSE =: Mini-Mental State Examination
MSD =: Meso Scale Diagnostics
NIA-AA =: National Institute on Aging-Alzheimer's Association
NPX =: Normalized Protein Expression
PEA =: Proximity Extension Assay
pTau =: phospho-tau
QC =: quality control
ROC =: receiver operating characteristic
SD =: standard deviation
