# Supplementary material for: Plasma biomarkers for prognosis of cognitive decline in patients with mild cognitive impairment
Source: Brain Commun. 2022 Jun 14;4(4):fcac155. doi: 10.1093/braincomms/fcac155 (PMC9257670; doi:10.1093/braincomms/fcac155)
Supplement: fcac155_Supplementary_Data [file fcac155_supplementary_data.zip › Supplementary Figure and Table Legends.docx]

**Supplementary Figure 1**. Correlation between plasma levels of NF-L measured by the Olink PEA technology and Quanterix Simoa. NF-L concentration was measured using the Quanterix Simoa NF-light Advantage Kit on a Simoa HD-1 analyzer.

**Supplementary Table 1**. Olink plasma extension assay (PEA) measurements of each participant.

**Supplementary Table 2.** Differential expression analysis of MCI-progressor vs. MCI-stable.

**Supplementary Table 3**. Differential expression analysis of Dem-AD vs. CN, Dem-Other vs. CN, and Dem-AD vs. Dem-Other.
